# Lymphatic Mesenteric Cyst, a Rare Cause of Surgical Abdominal Pain: Case Report and Review of the Literature

**DOI:** 10.7759/cureus.11766

**Published:** 2020-11-29

**Authors:** Bianca Cudia, Beatrice D'Orazio, Dario Calì, Gaetano Di Vita, Girolamo Geraci

**Affiliations:** 1 General Surgery Unit - Department of Surgical, Oncological and Stomatological Sciences, University of Palermo, Palermo, ITA

**Keywords:** mesenteric cyst, surgery, laparoscopy, abdominal pain

## Abstract

A lymphatic mesenteric cyst (LMC) is a rare clinical entity, of unclear etiopathogenesis, which can arise in the abdominal cavity or retroperitoneum without a clear origin.

We describe a case of a 74-year-old male presenting with abdominal pain that was non-specific and non-responsive to medical therapy. Laboratory tests clinical examination were inconclusive while the abdominal computed tomography (CT) scan showed a cystic lesion of the ileal mesentery. We performed an open surgical excision of the lesion with the resolution of clinical symptoms. The lesion resulted to be an LMC at the histological examination. At the five-year CT scan follow-up, we did not record any recurrences.

LMCs present without specific symptoms and imaging diagnostic techniques, such as ultrasound (US) or CT scan may define its features, location, or size. The preoperative diagnosis remains difficult, which is why the complete surgical excision is the gold standard treatment, aiming to prevent malignant transformation, complications, and recurrences.

## Introduction

The term “mesenteric cyst” (MC) refers to a heterogeneous group of cystic lesions with different etiopathogenetic roots, which appear in the abdominal cavity or in the retroperitoneum without knowledge of its abdominal organ of origin. The MC is a rare entity and its reported incidence ranges from one per 100.000 to one per 250.000 hospital admissions [1].

Several classifications for this kind of lesion have been proposed. In 2000, de Perrot et al. [2] introduced a classification, which nowadays is the most accepted, taking into account the histological features of the internal epithelium and resulting in six groups: lymphatic cyst, mesothelial cyst, enteric cyst, urogenital cyst, mature cyst teratoma, and pseudocyst. Paramythiotis et al., in 2018, performed a literature review finding only 16 cases that matched with MC in adults [3].

We present the case of a lymphatic mesenteric cyst (LMC) in a 74-year-old male patient coming under our observation.

## Case presentation

**Figure 1:**
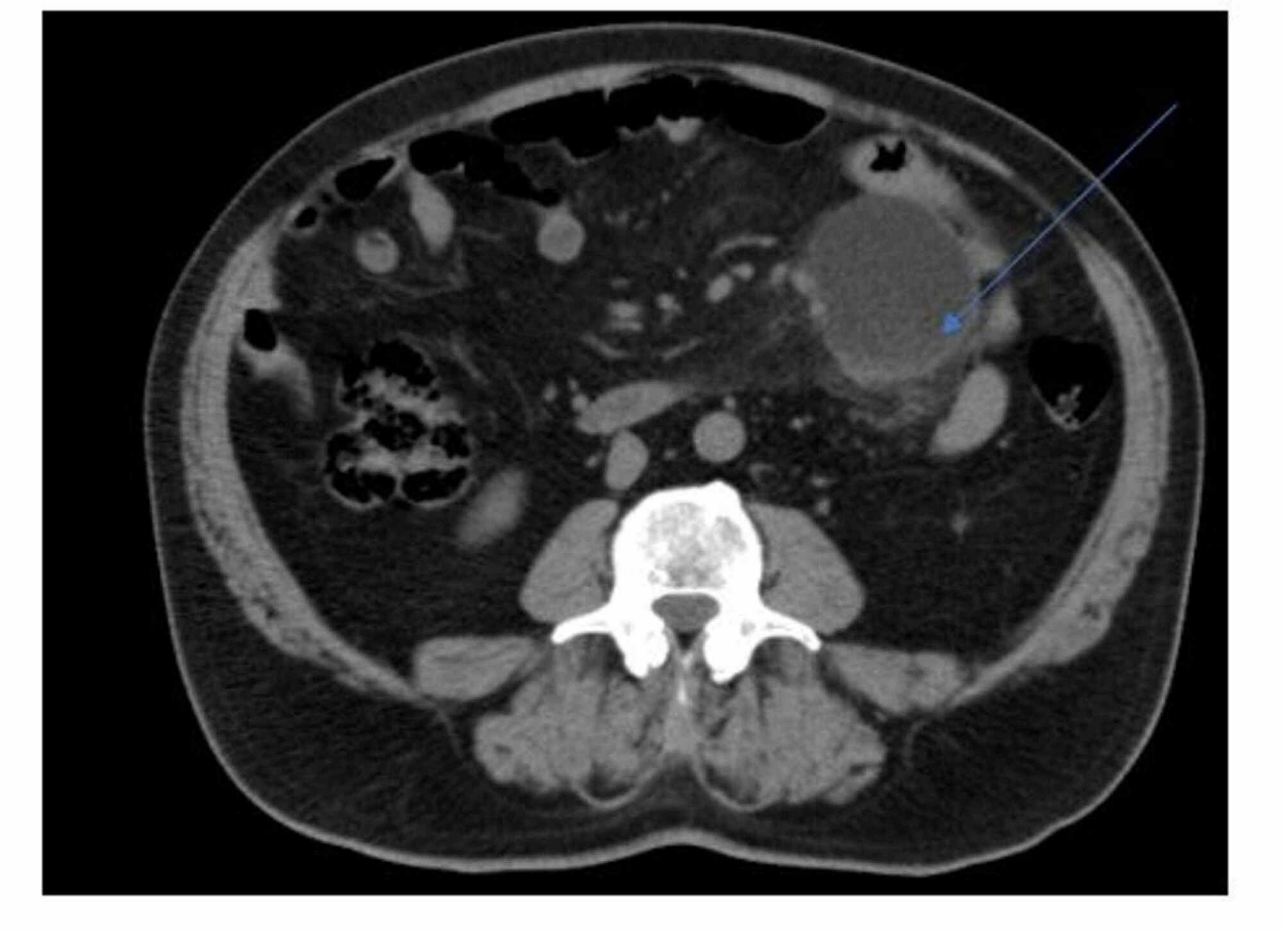
Abdominal CT scan frame Showing a cystic hypodense lesion with hyperdense content located between the mesenteric vessels in the left lower abdominal quadrant, which determines a dislocation of the intestinal loops and vessels along with hyperdensity of the surrounding fatty tissue. CT: computed tomography

A 74-year-old male patient presented with the appearance of diffuse abdominal pain for about two months without association with any other gastrointestinal (GI) or general symptoms. He has a personal history of essential hypertension without any other relevant fact. The physical examination showed only non-specific abdominal tenderness without signs of peritonitis and the laboratory blood tests were negative. We performed a complete abdominal ultrasound, which showed a cystic neoformation in the left lower abdominal quadrant with liquid content and did not detect any septae in it. A contrast-enhanced computed tomography (CT) scan of the abdomen was carried out (Figure 1). It showed a cystic hypodense mass of around 6x5 cm, with hyperdense content located between the mesenteric vessels in the left lower abdominal quadrant, which determined the displacement of the intestinal loops and vessels along with fat stranding, without contrast reinforcement and dissociable from the surrounding structures.

**Figure 2:**
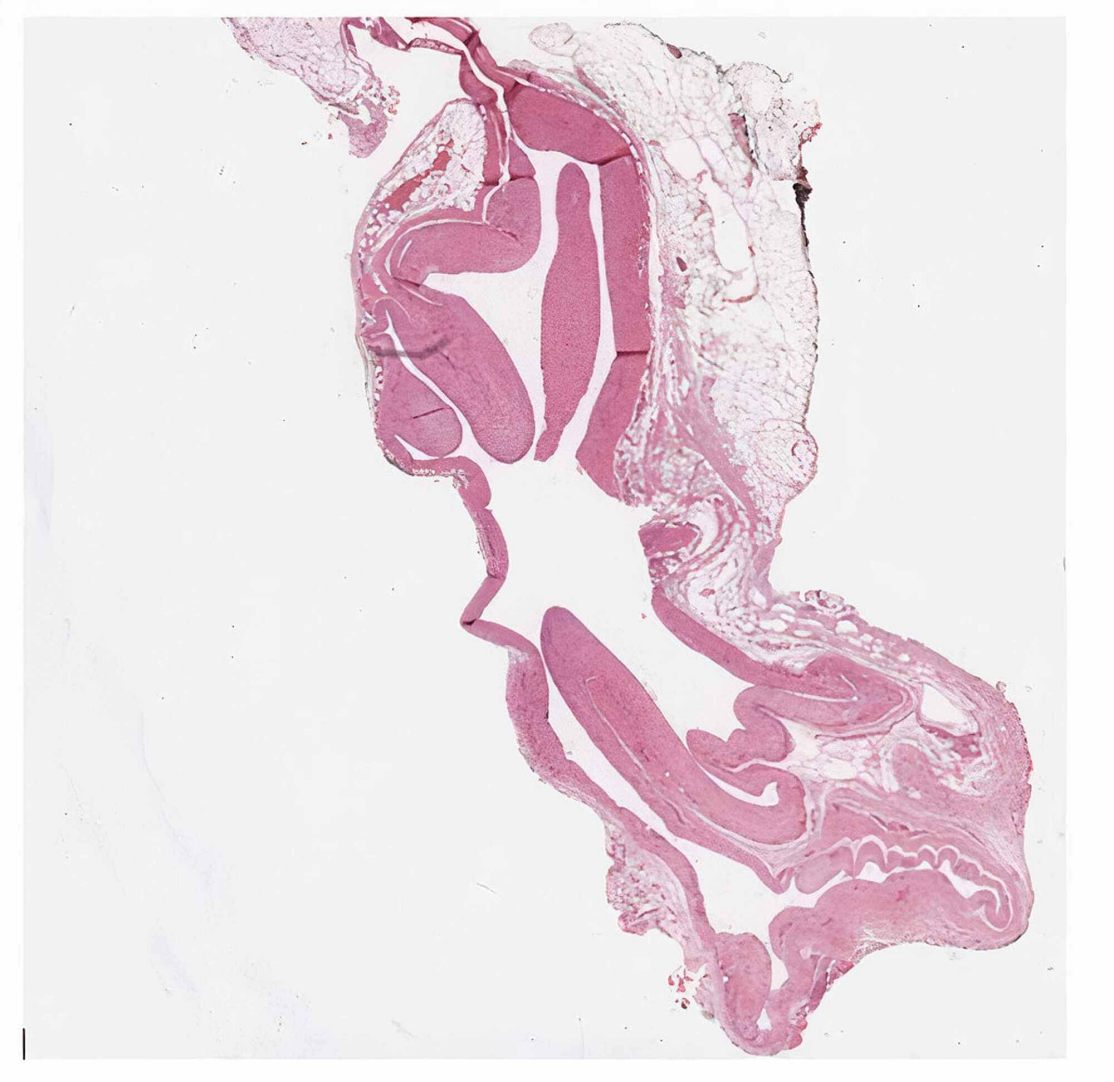
Hematoxylin-eosin, wholemount A lymphatic mesenteric cyst with serous content and a cystic lumen lined with endothelial cells of lymphatic subtype, flattened in a mono-stratified layer

We performed a complete open surgical excision of the lesion, which was located in the mesentery of the ileal loop. We did not have to perform an intestinal resection. The definitive histological examination pleads in favor of an LMC (Figure 2). In the immediate postoperative period, the clinical symptom of abdominal pain disappeared and the patient no longer experienced any other symptoms during the current follow-up. The postoperative course was uneventful, the patient was discharged on postoperative Day 7. At the five-year abdominal CT scan, we did not observe any recurrence.

## Discussion

LMC is defined as any cyst located in the mesentery with the possibility of being extended to the retroperitoneum. Usually, MC has a recognizable lining of endothelial or mesothelial cells [4] and can be located in any level of the gastrointestinal tract, most commonly arising from the ileal mesentery (60%) [5]. The first description of an MC has been reported from Benevieni, an Italian anatomist, who described it in 1507 in an atopic examination of an eight-year-old boy. It was Tillaux in 1880, who performed the first surgical excision of an MC.

Usually, MCs are unique lesions; in rare cases, they can be found as multiple ones. The male gender is more frequently involved. Their dimension of the MC may range from a few centimeters to 20 cm [6].

Histopathological examination of the surgical specimen may result in a unilocular or multilocular cyst containing serous or viscous fluid with chylomicrons, cholesterol crystals, and triglycerides surrounded by a single layer of flattened mesothelial immunoreactivity cells with cytokeratins and a fibrous wall with lymphocytes [3,7-9].

LMC etiology remains unknown but several mechanisms have been suggested to account for its development. The most commonly accepted theory is the benign proliferation of ectopic lymphatics in the mesentery that lack communication with the remainder of the lymphatic system. Another theory advocates the gradual enlargement of embryonic lymphatic channels, given the failure of venous system joining.

The clinical manifestation can be varied and non-specific. LMCs are often asymptomatic and occasionally diagnosed during diagnostic assessment for different diseases; they can cause non-specific abdominal pain or present themselves as a surgical emergency, determining complications such as bleeding, bowel infarction, or volvulus [4,10-11].

There must be a high diagnostic suspicion; although in most cases, the diagnosis is performed as an image finding. Ultrasound features may be various, but an MC should be considered in the presence of an avascular oval mesenteric mass; moreover, this diagnostic tool is superior to the CT in determining the nature of the cyst. A CT scan could be useful to exclude a super-infection, rupture, or internal bleeding by visualizing signs such as a thickened enhancing wall with perifocal fatty stranding. Also, ultrasound may detect this kind of complication underlining echogenic content, thickened capsule, or septations [11].

Magnetic resonance imaging can help the diagnosis by characterizing the origin and content of the MC, as well as pre-operative laparoscopy exploration.

The treatment of choice is their surgical excision in order to prevent a malignant transformation as well as the development of complications or recurrences. The preferred surgical technique should be laparoscopy and if the enucleation of the lesion cannot be performed safely, an intestinal loop resection “en bloc” may be necessary [12].

Even if good results have been achieved with percutaneous drainage and sclerosis of the abdominal wall, this management should be avoided for its high rate of recurrence [13-14].

## Conclusions

MCs are an extremely rare entity, given the lack of pathognomonic clinical, laboratory, and imaging features. Surgical excision is advisable in order to prevent the risk of complications, malignant transformation, and recurrences; moreover, the laparoscopic approach should be preferred.

## References

[1] Vanek VW, Phillips AK (1984). Retroperitoneal, mesenteric, and omental cysts. Arch Surg.

[2] de Perrot M, Bründler M, Tötsch M, Mentha G, Morel P (2000). Mesenteric cysts. Toward less confusion?. Dig Surg.

[3] Paramythiotis D, Bangeas P, Karakatsanis A, Iliadis A, Karayannopoulou G, Michalopoulos A (2018). Ideal treatment strategy for chylous mesenteric cyst: a case report. J Med Case Rep.

[4] Pithawa AK, Bansal AS, Kochar SP (2014). Mesenteric cyst: a rare intra-abdominal tumour. Med J Armed Forces India.

[5] Saviano MS, Fundarò S, Gelmini R, Begossi G, Perrone S, Farinetti A, Criscuolo M (1999). Mesenteric cystic neoformations: report of two cases. Surg Today.

[6] Aguirre SV, Mercedes Almagro M, Romero CA, Romero SS, Molina GA, Buenaño RA (2019). Giant mesenteric cyst from the small bowel mesentery in a young adult patient. J Surg Case Rep.

[7] Lee DL, Madhuvrata P, Reed MW, Balasubramanian SP (2016). Chylous mesenteric cyst: a diagnostic dilemma. Asian J Surg.

[8] Pantanowitz L, Botero M (2000). Giant mesenteric cyst: a case report and review of the literature. Int J Pathol.

[9] Javed A, Pal S, Chattopadhyay TK (2011). Chylolymphatic cysts of the mesentery. Trop Gastroenterol.

[10] Tan JJ, Tan KK, Chew SP (2009). Mesenteric cysts: an institution experience over 14 years and review of literature. World J Surg.

[11] Sudiono DR, Ponten JB, Zijta FM (2016). Acute abdominal pain caused by an infected mesenteric cyst in a 24-year-old female. Case Rep Radiol.

[12] Yasoshima T, Mukaiya M, Hirata K (2000). A chylous cyst of the mesentery: report of a case. Surg Today.

[13] Pozzi G, Ferrarese A, Borello A (2014). Percutaneous drainage and sclerosis of mesenteric cysts: literature overview and report of an innovative approach. Int J Surg.

[14] Ünlüer EE, Ünlüer S, Şahı N Y, Kamer KE, Karagöz A, Tan GC (2016). An uncommon cause of abdominal pain: mesenteric cyst. Interv Med Appl Sci.

